# Building a Diverse Workforce and Thinkforce to Reduce Health Disparities

**DOI:** 10.3390/ijerph18041569

**Published:** 2021-02-07

**Authors:** Richard Yanagihara, Marla J. Berry, Monica J. Carson, Sandra P. Chang, Heather Corliss, Marc B. Cox, Georges Haddad, Christine Hohmann, Scott T. Kelley, Eun Sook Yu Lee, Bruce G. Link, Richard J. Noel, Julie Pickrel, James T. Porter, Gregory J. Quirk, Temesgen Samuel, Jonathan K. Stiles, Angela U. Sy, Deborah A. Taira, Mary Jo Trepka, Fernando Villalta, Thomas E. Wiese

**Affiliations:** 1 University of Hawaii at Manoa, Honolulu, HI 96813, USA; mberry@hawaii.edu (M.J.B.); sandrac@hawaii.edu (S.P.C.); sya@hawaii.edu (A.U.S.); dtjuarez@hawaii.edu (D.A.T.); 2 University of California, Riverside, Riverside, CA 92521, USA; monica.carson@ucr.edu (M.J.C.); brucel@ucr.edu (B.G.L.); 3 San Diego State University, San Diego, CA 92182, USA; hcorliss@sdsu.edu (H.C.); skelley@sdsu.edu (S.T.K.); jpickrel@sdsu.edu (J.P.); 4 University of Texas at El Paso, El Paso, TX 79968, USA; mbcox@utep.edu; 5 Howard University, Washington, DC 20059, USA; ghaddad@howard.edu; 6 Morgan State University, Baltimore, MD 21251, USA; christine.hohmann@morgan.edu; 7 Florida Agricultural and Mechanical University, Tallahassee, FL 32307, USA; eunsook.lee@famu.edu; 8 Ponce Health Sciences University, Ponce, PR 00716, USA; rnoel@psm.edu (R.J.N.J.); jporter@psm.edu (J.T.P.); 9 University of Puerto Rico Medical Sciences Campus, San Juan, PR 00936, USA; gregoryjquirk@gmail.com; 10 Tuskegee University, Tuskegee, AL 36088, USA; tsamuel@tuskegee.edu; 11 Morehouse School of Medicine, Atlanta, GA 30310, USA; jstiles@msm.edu; 12 Florida International University, Miami, FL 33199, USA; trepkam@fiu.edu; 13 Meharry Medical College, Nashville, TN 37208, USA; fvillalta@mmc.edu; 14 Xavier University of Louisiana, New Orleans, LA 70125, USA; twiese@xula.edu

**Keywords:** RCMI, mentoring, workforce diversity, thinkforce heterogeneity, best practices

## Abstract

The Research Centers in Minority Institutions (RCMI) Program was congressionally mandated in 1985 to build research capacity at institutions that currently and historically recruit, train, and award doctorate degrees in the health professions and health-related sciences, primarily to individuals from underrepresented and minority populations. RCMI grantees share similar infrastructure needs and institutional goals. Of particular importance is the professional development of multidisciplinary teams of academic and community scholars (the “workforce”) and the harnessing of the heterogeneity of thought (the “thinkforce”) to reduce health disparities. The purpose of this report is to summarize the presentations and discussion at the RCMI Investigator Development Core (IDC) Workshop, held in conjunction with the RCMI Program National Conference in Bethesda, Maryland, in December 2019. The RCMI IDC Directors provided information about their professional development activities and Pilot Projects Programs and discussed barriers identified by new and early-stage investigators that limit effective career development, as well as potential solutions to overcome such obstacles. This report also proposes potential alignments of professional development activities, targeted goals and common metrics to track productivity and success.

## 1. Introduction

Achieving long-term health and wellness in communities that suffer disproportionately from racial, ethnic, environmental, and social and/or economic inequalities and inequities demands building institutional capacity and enhancing infrastructure on many levels. Particularly at institutions of higher education and health-professions schools, a major component of this infrastructure is the professional development of diverse teams of academic and community scholars (the “workforce”), who understand the challenges faced by underserved and vulnerable populations because of shared life experiences (the “thinkforce”), to collaboratively explore and identify the causes of health disparities, to design and test the best measures and methods for studying health disparities, and to develop and implement the most effective and culturally appropriate interventions for reducing and eliminating health disparities [1,2,3]. Funding from the Research Centers in Minority Institutions (RCMI) Program has begun to transform formerly resource-limited institutions of higher education and health-professions schools to have a seat at the table as equal partners with research-intensive majority institutions to accelerate the acquisition of new knowledge to improve the health of the nation [4,5].

### 1.1. Brief History of the RCMI Program

House Report 98-911, attached to H.R. 6028 (of the Departments of Labor, Health and Human Services, and Education and Related Agencies Appropriation Act, 1985), provided the original language to establish research centers in predominantly minority institutions, which offered doctoral degrees in the health professions and/or the health-related sciences to individuals from underrepresented, underserved, and minority populations [5]. Subsequent legislation (H.R. 3010) further recognized the critical role played by these institutions in delivering healthcare services to medically underserved communities.

Originally administered by the National Center for Research Resources (NCRR), of the National Institutes of Health (NIH), the RCMI Program provided support to two inaugural institutions: Florida Agricultural and Mechanical University (FAMU) and Ponce School of Medicine (PHSU). The next cohort, in 1986, included Clark Atlanta University, Meharry Medical College (MMC), University of Hawaii at Manoa (UHM), and University of Puerto Rico Medical Sciences Campus (UPR-MSC), followed in 1987 by Howard University (HU) and Morehouse School of Medicine (MSM). The annual operating budget of the fledgling RCMI Program, initially less than $1 million, has grown to more than $74 million in FY2020. Such growth has been justified by the successful leveraging of RCMI funds by RCMI grantee institutions and supported through strong congressional advocacy.

As part of the NIH reorganization to create the National Center for Advancing Translational Sciences (NCATS), NCRR was dismantled in 2011 [6], and the RCMI Program was transferred to the National Institute on Minority Health and Health Disparities (NIMHD). Beginning in 2017, the various RCMI funding mechanisms have been consolidated and transitioned to a single U54 cooperative agreement program, focusing on advancing research on the science of minority health and health disparities. This has extended the geographic reach of the RCMI Program to include new centers at Florida International University (FIU), Morgan State University (MSU), North Carolina Central University, Northern Arizona University, San Diego State University (SDSU), University of California, Riverside (UCR), and University of Houston.

### 1.2. RCMI Specialized Centers

A principal goal of the RCMI Specialized Centers is to create environments conducive to career enhancement for all investigators, and particularly women, persons with disabilities, and those from racial and ethnic groups which are underrepresented in biomedical research, as defined by the National Science Foundation (i.e., African Americans or Blacks, Hispanics, American Indians and Alaska Natives, and Native Hawaiians and other Pacific Islanders). These environments are designed to assist investigators in becoming more successful in obtaining competitive extramural funding for basic biomedical, behavioral, clinical and/or population health research on diseases and conditions that disproportionately affect the health and well-being of ethnic minority and other underserved and marginalized communities, including the poor, homeless, rural persons, sexual and gender minorities, and recent immigrants. Another goal of the RCMI Centers is to strengthen and sustain partnerships with community-based and other non-academic organizations that reach the intended underserved populations to collaborate on the dissemination and implementation of culturally appropriate and contextually relevant interventions for health promotion and disease prevention. In short, the RCMI Centers are meant to diversify the workforce, enhance the quality of scientific inquiry, and promote collaborative research that improves minority health and reduces long-standing health disparities.

As instructed in the funding opportunity announcements RFA-MD-17-003, RFA-MD-17-006, RFA-MD-18-018, issued by NIMHD, each RCMI Specialized Center had to be structured with four distinct cores: Administrative Core (AC), Community Engagement Core (CEC), Investigator Development Core (IDC), and Research Infrastructure Core (RIC). The primary goal of the IDC is to create a supportive career development milieu for post-doctoral fellows, junior faculty, and other early-stage investigators to conduct hypothesis-driven basic biomedical, behavioral, and/or clinical research that results in significant return on investment, as measured by peer-reviewed publications in high-impact journals and successful NIH grant applications, among other metrics. The IDC is also expected to develop and implement a Pilot Projects Program to provide funding on a competitive basis to new and junior investigators.

The shared goals and expected functions of the RCMI IDC provide an unparalleled opportunity to improve coordination, increase efficiency and leverage resources for professional development across all RCMI Centers. Accordingly, the primary objective of the RCMI IDC Workshop, held in conjunction with the RCMI Program National Conference in Bethesda, Maryland, in December 2019, was for IDC Directors to meet each other and to learn first-hand about the professional development activities and Pilot Projects Program at each RCMI Center with the goal of stimulating problem-solving discussions around common challenges to strengthen each RCMI Center and the collective RCMI Program. The near-term desired outcome was relationship building to facilitate the development of an IDC Consortium, comprising RCMI IDC Directors, which would meet regularly to enhance resource sharing to build a diverse workforce and thinkforce to reduce health disparities.

## 2. Workshop Planning

The logistics and organization of the IDC Workshop were handled by the Research Coordinating Center, of the former RCMI Translational Research Network [4,5], and the workshop agenda was developed with input from the IDC Directors and an RCMI Program Official from NIMHD. The workshop was divided into three distinct sessions and was designed to maximize information sharing and to foster face-to-face interactions among IDC Directors, or designees, with the added goal of preparing a report for the conference proceedings.

The workshop started with an Information-Sharing Session to allow each Center to present a brief summary of their IDC activities, focusing primarily on professional development activities and on the Pilot Projects Program. Because the RCMI Centers have different project start dates, and because some RCMI Centers were transitioning from funding through the prior G12 funding mechanism while other RCMI Centers had no previous RCMI funding history, some Centers had data about the number of Pilot Projects Program project proposals received and reviewed, whereas other Centers were limited to presenting their plans. Nevertheless, the final slide of each presentation was devoted to articulating challenges and barriers to effective execution of IDC activities.

Next, during the Problem-Solving Session, the IDC Directors and other workshop participants discussed the challenges and barriers encountered in implementing mentoring efforts and the Pilot Projects Program. Participants also shared potential solutions, and discussed milestones and common metrics to better track progress and to arrive at best practices and harmonization.

Both the Information-Sharing Session and the Problem-Solving Session were informed by data collected from RCMI Investigator Needs Assessment Surveys, previously performed by the RTRN Research Coordinating Core. That is, the barriers and obstacles to optimal mentoring and professional development, as well as potential solutions, were those articulated by basic biomedical, behavioral, and clinical researchers at all academic levels, including junior investigators, who played a significant role in providing input through their real-life experiences.

Finally, during the Networking and Evaluation Session, participants completed an evaluation survey about the workshop and were allowed time for unstructured interactions and relationship building to form the basis for the establishment of the envisioned IDC Consortium and sustained engagement of IDC Directors to achieve shared goals.

## 3. Workshop Implementation

The IDC Workshop was well attended, with active participation from 14 RCMI Centers: FAMU, FIU, HU, MMC, MSM, MSU, PHSU, SDSU, Tuskegee University (TU), UCR, UHM, UPR-MSC, University of Texas at El Paso (UTEP), and Xavier University of Louisiana (XULA). Each Center provided an overview of their mentoring and investigator development activities, as well as Pilot Projects Program.

### 3.1. Professional Development Activities

Common features were evident in the IDC of each RCMI Center. Specifically, the IDC provided personalized mentoring in scientific and career development to junior and early-stage investigators, as well as senior postdoctoral fellows. The IDC Directors acknowledged that effective mentoring was a key factor to successful career development. However, the process of identifying promising junior investigators and matching them with mentors who are compatible in terms of their personality, temperament, scientific knowledge, history of NIH funding, and mentoring skills is not fool proof and requires thoughtful, personalized attention. Research networking tools, such as the open-source Profiles Research Networking Software originally developed by Harvard Medical School and enhanced by the University of California San Francisco [7], can provide current information on possible scientific mentors. However, developing registries or databases of mentors will not in and of themselves result in suitable and sustainable mentoring relationships. IDC Directors acknowledged that one of their primary responsibilities was to assist mentees in making the best selections and in establishing and cultivating strong working relationships with prospective mentors, especially those from outside institutions, as well as to secure commitments to regularly scheduled meetings.

Investigator development activities at nearly all RCMI Centers consisted of developing mentoring teams, providing grant writing workshops, and arranging seminars, featuring internal and external researchers speaking about their research and topics related to health disparities and professional development. Importantly, junior investigators were provided opportunities to meet with speakers to foster mentorship and collaboration.

The HU IDC presented a systematic and methodological program to fast track the development of junior faculty and early-stage investigators, using a tri-faceted approach that involved providing pilot seed-grant support, coordinating comprehensive career mentoring that included both discipline-specific research and professional advancement mentoring, and offering professional development trainings relevant to early-stage investigators (e.g., grantsmanship, scientific presentation skills, and the elements and processes involved in scientific writing).

At FIU, mentoring activities were largely outcomes-driven and closely linked with the Pilot Projects Program. Recipients of pilot funding met regularly with RCMI faculty and other awardees for advice and support, with linkages to RCMI research training, RCMI research opportunities, RCMI community connections, and career development activities through the RCMI and other university-wide programs.

Similarly, the FAMU IDC and MMC IDC focused mentoring efforts and other professional development activities and resources on recipients of pilot funding. MMC investigators were able to request a Studio [8,9], where expert scientists reviewed specific aims, research design and/or community engagement. Further, mock study section reviews were available to MMC investigators through the Vanderbilt Institute of Clinical and Translational Research (VICTR). FAMU IDC provided personalized mentoring and workshops for investigators to compete successfully for extramural funding.

The SDSU IDC provides two avenues for career enhancement: a year-long program to awardees of pilot project funding, and open-forum career enhancement activities for all interested parties. For pilot project awardees, the SDSU IDC offers highly structured monthly mentoring sessions that cover wide-ranging topics, including individual development plans, project management, data management and safety, and optimizing success. The open-forum career enhancement activities generally focus on a specific topic or skill, such as how to select an appropriate electronic data capture system, how to effectively partner with external organizations on minority health and health disparities research, among others.

The MSM IDC was built on the foundation of their well-developed Mentoring Academy [10] that worked in synergy with the AC, CEC, and RIC to create a nurturing environment for the next generation of biomedical investigators to achieve the mission of advancing health equity. Research Studios and intensive coaching of early-stage investigators to prepare and submit NIH grant applications through the National Research Mentoring Network (NRMN) [11] were key features.

The MSU IDC provided a summer mini-sabbatical for its RCMI investigators to build collaborations at partnering institutions in Baltimore, such as the University of Maryland and Johns Hopkins University School of Medicine.

The PHSU IDC provided a two-year program, known as Strategic Academic Research Training (START), to approximately six scholars per year. Emphasis was placed on enhancing the diversity that their scholars brought to the scientific workforce.

- At TU, mentoring and professional development activities were focused on early-stage investigators. In addition, the IDC was intent on supporting senior or established investigators, who were transitioning into research on the science of health disparities from non-biomedical fields or biomedical fields not directly related to minority health and health disparities.

The UCR IDC’s Big Feasible and Fundable Seminar Series was linked with the AC’s Interdisciplinary Research Workgroups to create pipelines for identifying and recruiting junior investigators and to facilitate their acquisition of essential skills in communicating and collaborating across disparate disciplines. That is, Individual Multidisciplinary Mentorship Teams comprised mentors from within the trainee’s discipline and from complementary disciplines.

- At UPR-MSC, IDC mentoring activities included informal meetings, rehearsal of scientific presentations, discussion of topics relevant to successful transition to running a lab (using the best-seller book: “At the Helm: Leading your Laboratory”), and mock study sections. Trainees regularly review their five-year plans for grants, publications, and promotion.
- At UTEP, the IDC uses a “synergy = success” model, which incorporated the commitments of multiple stakeholders at the university, programmatic, and external levels to ensure sustained support of RCMI investigators. Apart from intensive training on grant writing, course release was offered to investigators in exchange for preparing and submitting grant applications, and senior postdoctoral fellows and senior investigators were provided opportunities to secure short-term starter funds and bridge funds, respectively.
- At XULA, the matching of junior investigators with collaborators and mentors occurred even before new faculty were officially recruited and multiple activities linked early-stage investigators, mid-career faculty and research scientists to RCMI and non-RCMI resources.

Professional development activities for new and early-stage investigators at RCMI Centers can be generally grouped into three categories: Professional Conduct, Research Resources, and Career Skills and Knowledge. As an illustration, Table 1 lists the principal topics covered in each category by the month-long Team-Science Mentoring Bootcamp, offered annually by the UHM RCMI Center. The sessions have been well attended by junior faculty from diverse disciplines and have attracted faculty from several other RCMI Centers through the online Zoom platform. Further efforts to provide greater access to such professional development activities across the network of RCMI grantee institutions are expected to promote the formation of cross-institutional mentoring teams, as well as the sharing of resources for multi-center research collaborations.

### 3.2. Barriers and Obstacles to Professional Development

Through input from junior faculty at their institutions, the IDC Directors reported multiple barriers and obstacles to professional development (Table 2). Among the most common barrier was the paucity of NIH-funded senior faculty, who could serve as role models and mentors to early-stage RCMI investigators. Having extramural funding, however, did not ensure that such senior faculty had the necessary mentoring experience and/or skills to create suitably supportive environments for junior faculty. In fact, some senior faculty were never properly mentored and some tended to have an inappropriately overbearing mentoring style and failed to embrace differences in thought and thinking, thereby delaying the development of junior investigators into independently funded researchers. As emphasized in a recent National Academies of Sciences, Engineering, and Medicine report, mentoring has too often been left to occur organically or by chance, and has not received the “focused attention, evaluation, and recognition of other aspects of professional development, such as teaching and research” [12]. Accordingly, far more effort needs to be directed toward providing opportunities for senior and mid-level faculty to learn mentoring skills [13]. Investments in this type of mentoring-the-mentor initiatives would also have a multiplicative effect in providing junior faculty with mentoring skills.

In addition to needing compatible mentors, junior faculty frequently mentioned other barriers to professional development, including their lack of knowledge about finding funding and training opportunities; the lack of pre-award grant application preparation; the lack of internal grants review system; the lack of bridging funds to continue while awaiting funding decisions.

Apart from individual barriers to professional development, there were institutional obstacles that limited the growth of a diversified workforce for research on the science of minority health and health disparities at RCMI Centers. For example, institutional hiring freezes, limited institutional start-up funds for newly recruited faculty, insufficient grants development support, high administrative leadership turnover, and lack of positions for postdoctoral fellows in the absence of training grants, both individually and collectively, stifle the growth of a critical mass of investigators in specific research areas. In addition, broken promises and unfulfilled commitments by institutional leadership have occurred because of changes in organizational structure and/or changes in administration, as well as downturn in financial wellbeing.

### 3.3. Solutions to Reduce Barriers to Professional Development

For each barrier and obstacle, potential solutions and enablers were identified to optimize mentoring and professional development of new and early-stage RCMI investigators (Table 2). A possible solution to the paucity of NIH-funded senior faculty is to enlist mentors from non-RCMI grantee institutions and to enhance the searchable Profiles database of faculty across the RCMI Centers. Grouping such RCMI faculty according to research category (basic biomedical, behavioral, and clinical), as well as by the NIH Institutes and Centers (ICs) providing their support, would facilitate suitable mentee-mentor matches. The IDC Directors and IDC staff, with the assistance of the RCMI Coordinating Center, must take responsibility for guiding emerging investigators to use the Profiles database to build their mentoring and collaborative investigative teams.

### 3.4. Alignment of Professional Development Activities

Coordinating and leveraging the IDC infrastructure of the RCMI Centers, as well as engaging non-RCMI programs, including the Clinical and Translational Science Awards (CTSA), Institutional Development Award (IDeA), Centers for AIDS Research (CFAR), and Transdisciplinary Collaborative Centers (TCC), to serve on mentoring teams will accelerate attainment of research independence of early-stage RCMI investigators. Intensified engagement with the RCMI IDC Directors and the formation of an IDC Consortium would facilitate better alignment of professional development activities across the RCMI Centers (Table 3). For example, online grant writing workshops held at RCMI Centers might be made available broadly across the RCMI Centers, like the intensive coaching programs such as NRMN. Furthermore, online sessions for peer mentoring could be scheduled to allow early-stage RCMI investigators to brainstorm and learn from one another in a non-threatening forum. Apart from finding well-suited mentors for junior investigators, the IDC Consortium should collaborate closely with the RIC Consortium and CEC Consortium to maximize use of core facilities and widen access to vulnerable communities across RCMI Centers.

### 3.5. Pilot Projects Programs

The IDC of each RCMI Center is expected to develop and manage a Pilot Projects Program to provide funding through a competitive process to RCMI investigators. Inherent in this approach are the expected outcomes of accelerating the pace of scientific discovery, heightening research productivity, increasing competitiveness for mainstream extramural funding, and ultimately improving the health of the nation. Currently, the awards for such pilot projects have ranged between $30,000 and $50,000 per year. For most RCMI Centers, the funding period for pilot projects was one year, but in the case of UPR-MSC, XULA, and SDSU, two or more years of support were granted. More than one year of funding was deemed especially necessary for community-engaged research projects. Thus far, the number of awards has varied from two to five pilot projects per year. However, insufficient data were available from many of the Pilot Projects Programs to assess success or return on investment.

The FIU Pilot Projects Program planned to fund up to five pilot projects per year, with a goal of up to 20 projects during the five-year funding cycle. To date, eight FIU junior faculty have been funded, and each is preparing an NIH grant application (1 R01; 1 R21; 6 K series).

The UCR Pilot Projects Program provides support through four mechanisms: (1) Fostering Interdisciplinary Research-early STage Awards (FIRST Awards) serve as transition awards aimed at increasing competitiveness at obtaining external funding; (2) Pilot Interdisciplinary Collaborative grants (PIC Grants) seek to support goals of interdisciplinary research working groups; (3) Interdisciplinary Research Work Group Awards (IRWG Award) funds problem-focused interdisciplinary health disparities research; and (4) Continuity Collaborative Fellowships are awarded twice a year to fill gaps in research and database management in ongoing community-based research projects, conducted by graduate students or postdoctoral fellows.

The XULA Pilot Projects Program, which focuses on early-stage investigators engaged in clinical and population research involving cancer health disparities, solicits applications jointly with the Louisiana Cancer Research Center (LCRC) and Project Pathways, of the Building Infrastructure Leading to Diversity (BUILD) program. Of 16 pilot applications submitted in 2019, three were funded through RCMI, three with LCRC, and six by BUILD.

The MSM Pilot Projects Program involves a letter-of-intent. In 2019, full applications were invited from five of 11 investigators who originally submitted a letter-of-intent, and two awards were made. Applicants who were not funded were encouraged by the IDC to revise and resubmit their applications to the next round or to submit to other funding agencies.

The UHM Pilot Projects Program also has a two-phase process. In the first phase, one-page abstracts and NIH Biosketches of the applicant(s), mentors and collaborators are reviewed by the principal investigators and core directors. In the second phase, invited full applications are evaluated by outside reviewers. Over three funding cycles, 83 abstracts were received and 37 full applications were invited, from which 15 pilot awards were made, resulting in 71 publications and 34 new grants (totaling more than $4 million, or a return on investment of 6.3).

The HU Pilot Projects Program has had similar outcomes from seven pilot awardees, who have been responsible for 31 publications and 11 new grants, with a return on investment of 6.4 over two funding cycles.

### 3.6. Barriers and Obstacles to Pilot Projects Programs

The RCMI Centers identified multiple barriers to the effective implementation of the Pilot Projects Program (Table 4). Among the most common barrier was the dearth of subject-matter experts with NIH study section experience who could serve as reviewers. Further, real or perceived conflicts of interest were commonplace at RCMI Centers because of the limited number of faculty in some research areas. Another major barrier was that recipients of pilot funding became ineligible for the Support of Competitive Research (SCORE) Program, thus reducing the pool of promising prospective applicants. Institutional barriers, such as delays in the review and approval processes for human subjects and vertebrate animal research, heavy teaching loads for junior faculty, and insufficient protected time for clinicians, tended to be more daunting obstacles to creating a robust applicant pool and sustaining a vibrant Pilot Projects Program.

### 3.7. Solutions to Reduce Barriers to Pilot Projects Programs

Potential solutions were identified to optimize the implementation of the Pilot Projects Program at RCMI Centers (Table 4). As in mentoring, a robust database of RCMI faculty with NIH study section experience to serve as reviewers would help to overcome the limited reviewer pool and conflicts of interest at individual RCMI Centers. Similarly, the sharing of expertise within the RIC and CEC would address research support needs of biostatistics, bioinformatics, study design, community engagement, and recruitment and retention. Conceivably, these individuals from the RIC and CEC might provide assistance with study design, initial scientific review, trouble shooting of technical issues, and assessment of feasibility. This type of resource sharing of human capital would strengthen individual RCMI Centers and the overall RCMI Program.

Heavy teaching loads for junior faculty and insufficient protected time for clinicians served as major deterrents to preparing proposals for Pilot Projects Programs. Finding solutions to these barriers is not a simple matter. Institutional investments are required at the department and college or school level. Particularly for newly hired basic scientists, a one- or two-year postponement of teaching obligations, coupled with intensive mentoring, would heighten the probability of grant success. Similarly, providing several months-long periods of protected time for clinical faculty would likely boost the number of grant proposals submitted and the likelihood of success, and once such faculty garner grant support, their mentors and IDC Directors should serve as strong advocates to negotiate reductions in teaching loads and clinical assignments.

### 3.8. Alignment of Pilot Projects Programs

Alignment or harmonization of the processes and procedures of the Pilot Projects Programs at RCMI Centers would result in the achievement of specific goals each year (Table 5). For example, the sharing of faculty to serve as reviewers might result in the creation of a pre-submission internal grants review system for RCMI investigators submitting NIH applications. Additionally, individual IDC Directors might learn from other RCMI Centers about how to better streamline the solicitation, receipt and review processes of their Pilot Projects Programs. For example, the UHM RCMI Center has successfully developed and deployed a user-friendly online submission and review system, which could be adapted by other RCMI Centers. Likewise, the SDSU RCMI Center was able to adopt a system used by the university for its intramural grant opportunities.

Continued assistance to RCMI investigators whose applications were not selected for funding would constitute another way to align Pilot Projects Programs. That is, the professional development function of the IDC Consortium should be inextricably linked with the Pilot Projects Program function. The annual outcomes and goals in Table 5 are tentative and further discussion will be necessary before full adoption by the IDC Consortium, with periodic review and modification. Each RCMI IDC might also wish to establish additional institution-specific annual outcomes for their pilot project awardees. In either case, clear articulation of the annual outcomes and goals must be hard-wired into the Pilot Projects Program.

## 4. Discussion

The opportunity for IDC Directors to meet face-to-face, nearly all for the first time, and to share their experiences, as well as to discuss their challenges at the IDC Workshop, has laid the foundation for the creation of an IDC Consortium, comprising the IDC Directors from RCMI Centers, to work collaboratively to overcome obstacles in professional development activities and to promote greater efficiency in the review process of pilot project proposals. The newly funded RCMI Coordinating Center will serve as a communications hub to facilitate the scheduling of regular meetings of the IDC Consortium and to coordinate continued interactions, resource sharing and problem solving to achieve the collective goals of advancing the careers of underrepresented minority investigators and those conducting minority health and health disparities research.

By leveraging the leadership, expertise and talent of the RCMI IDC Consortium, there is every expectation that performance outcomes will improve and productivity measures will increase at individual RCMI Centers and in the overall RCMI Program. Table 6 provides key IDC activities and key metrics common to all RCMI Centers for tracking and evaluation of professional development activities and how activities and metrics align with and contribute to goals of the RCMI Program. Professional development activities and the Pilot Projects Programs of the RCMI IDC will focus on identifying the causes of health disparities, on determining the best methodologies for measuring health disparities, and on developing and implementing culturally appropriate interventions to reduce health disparities. Regularly scheduled meetings of the IDC Consortium would provide a forum to modify common metrics and to work toward achieving shared goals, as well as to harmonize activities with the RIC Consortium and CEC Consortium. In addition, the IDC Consortium will collaborate closely with the RCMI evaluators [14].

Although there are institution-specific barriers to building research capacity [15], there are also many similarities across all RCMI Centers. Thus, the sharing of solutions that have worked at individual RCMI Centers could have a powerful impact on other RCMI Centers. In this regard, creating a compendium of best practices for pilot projects programs [16] and for professional development activities would be valuable for the IDC Consortium. Recently, a literature review of 46 papers published in English from 2010 to 2020 examined the barriers and facilitators to mentoring of new and early-stage investigators, as well as underrepresented minority faculty in health-related research [17]. Lack of time, lack of mentors, lack of access to resources, and heavy teaching and service loads were the most frequently mentioned barriers to achieving success in health-related research. These were also prominently featured by RCMI IDC Directors at the workshop.

Junior RCMI faculty do not typically encounter institutional racism, discrimination and/or bias, in large part because all RCMI grantee institutions serve predominantly racial and ethnic minorities and individuals from disadvantaged socio-economic backgrounds, and because many senior RCMI faculty and IDC Directors have themselves experienced hardships and barriers and understand the challenges faced by junior faculty. The lack of generational pipelines and access to academia is a well-known barrier to success. While not mentioned or discussed during the workshop, persistent barriers to professional development in the Science, Technology, Engineering, Mathematics, and Medicine (STEMM) pipeline perpetuate racial disparities in academia. Solutions lie in earlier exposure and access to resources, supportive mentoring networks, and comprehensive training programs for racial and ethnic minority students and trainees at each career stage [18]. Multiple NIH programs have been initiated and expanded to provide more research training opportunities to ethnic minority and disadvantaged students [19]. However, this “leaky pipeline” extends into later stages of career development, particularly for women [20,21].

In addition to monthly IDC Consortium-wide meetings, periodic regional meetings based on geography and time zones might further strengthen relationship building, enhance resource sharing, and facilitate and expedite the review of pilot project proposals. Currently, there are 21 RCMI Centers, distributed in 12 states, the District of Columbia, and Puerto Rico. Each regional sub-consortium could comprise four or more RCMI IDC (Table 7), and the host of regional meetings would rotate. Using this regional organizational structure for the RIC Consortium and CEC Consortium might lead to improved overall networking and would leverage the sharing of core facilities, foster collaborations, and streamline coordination across RCMI Centers.

Professional development activities are also enhanced through resources within the RIC and CEC of each RCMI Center, as well as leveraged through considerable resources available in collaborations and partnerships with other NIH-funded infrastructure programs, such as CTSA, IDeA, CFAR, and TCC, at non-RCMI grantee institutions [22,23]. Moreover, these partnerships provide RCMI investigators with access to pilot funding. Examples include partnerships between MSM with the Emory CFAR, and UHM with the University of Washington CFAR; partnerships between XULA with the Louisiana CTSA, MMC with VICTR, and MSM with the Georgia CTSA. HU and Georgetown serve as equal partners in a CTSA, known as the Georgetown-Howard Universities Center for Clinical and Translational Science (GHUCCTS).

## 5. Conclusions

Community-driven and culturally relevant solutions to eliminating health disparities necessitate the creation of a supportive mentoring milieu to accelerate the career development of diverse and multidisciplinary teams of scholars (the “workforce”) and to harness the heterogeneity of their thought and thinking (the “thinkforce”). That is, only by embracing and celebrating the power of different perspectives, tempered by unique lived experiences and influenced by cultural and indigenous beliefs, as well as age-old community practices and norms, can one expect to pose previously unasked research questions and to design distinctly alternative approaches that lead to innovative interpretations of data and sustainable strategies for disease prevention and health promotion for underserved populations and marginalized communities. The IDC Consortium provides an exciting platform for productive interactions that foster the sharing of research resources and human capital across the RCMI Centers to hasten the acquisition of new knowledge to improve minority health and to reduce health disparities. Such interactions are also likely to strengthen and sustain inter-institutional collaborations and partnerships with non-RCMI grantee institutions [22,23].

## Figures and Tables

**Table 1:** Professional Development Team-Science Bootcamp at the University of Hawaii at Manoa (UHM). Research Centers in Minority Institutions (RCMI) Center.

Category	Representative Topics
Professional Conduct	Research integrity and professional ethics Responsible conduct of research Research administration: financial and personnel management Materials, ownership, and materials transfer agreements Intellectual property, copyrights, patents, licenses, technology transfer Conflicts of interest
Research Resources	Research design; biostatistics; bioinformatics; data acquisition, management and analysis Research involving human subjects or tissues; regulatory and compliance issues Research involving vertebrate animals; regulatory and compliance issues Environmental safety: radiation, chemicals, biological/select agents Survey design and administration; dissemination and implementation
Career Skills and Knowledge	Community-engaged research and community-based participatory research tutorials and workshops Principles and best practices for building trust-based relationships with communities Mentor and mentee responsibilities, collaborative team science Time management and working strategically Authorship and publication practices, navigating collaborative publications Manuscript preparation, submission and revisions Public-speaking, presentation and communication skills Identifying funding opportunities, understanding different NIH funding mechanisms, grant writing and grantsmanship, grant submission Promotion and tenure dossier preparation Career opportunities in academia, industry, state and federal government, and global organizations

**Table 2:** Barriers to Professional Development and Potential Solutions.

Barriers	Potential Solutions
Dearth of NIH-funded RCMI faculty to serve as mentors	Update Profiles database of NIH-funded RCMI faculty
Few role models of ethnic- and gender-appropriate NIH-funded mentors	Create mentoring teams comprising compatible and responsive senior faculty
Paucity of well-trained senior faculty to provide technical training in specific areas	Enlist RCMI and non-RCMI faculty to provide targeted training
Overburdened senior faculty with inadequate time and/or ability to serve as mentors	Incentivize mentoring and cultivate mentoring skills in senior faculty
Rigid one-size-fits-all mentoring philosophy	Promote individualized or personalized best-fit mentoring philosophy
Insufficient exposure to first-tier researchers	Invite world-class RCMI and non-RCMI scholars to meet with junior faculty
Inadequate mechanism to support faculty while awaiting funding decision	Provide bridging funds to faculty awaiting funding decisions on grant applications
No internal review mechanism of grant applications	Create internal review mechanism to increase grant success
Lack of knowledge about funding opportunities and grantsmanship	Sponsor funding tutorials and grantsmanship workshops
Limited research training opportunities	Expand training opportunities by partnering with RCMI and non-RCMI grantee institutions
Limited support for pre-award grant development	Leverage RCMI funding to gain institutional commitment for grants development support
Broken promises and unfulfilled commitments	Leverage External Advisory Committee

**Table 3:** Alignment of Professional Development Activities.

Processes and Procedures	Outputs and Deliverables	Annual Outcomes and Goals
Develop registry of NIH-funded RCMI mentors with expertise in areas related to NIH ICs Create mentoring teams Match mentees and mentors from different RCMI Centers Assist mentees in preparing manuscripts and grant Applications	Prepare manual of best practices and standard operating procedures for mentoring and professional development Prepare a webinar series on career skills and knowledge Launch program for early-stage investigators to train at another RCMI Center	Demonstrate at least one cross-mentored or joint grant application per institution Demonstrate at least one joint publication by faculty at different RCMI Centers Demonstrate at least one nomination for membership into a scientific society per institution initiated by faculty from different RCMI Centers

**Table 4:** Barriers to Pilot Projects Programs and Potential Solutions.

Barriers	Potential Solutions
Insufficient subject-matter experts with NIH study section experience to serve as reviewers	Create registry of subject-matter experts with NIH study section experience across RCMI Centers
Real or perceived conflict of interest among available reviewers	Enlist faculty from other RCMI Centers and External Advisors as reviewers
Paper-based submission and review process	Adopt on-line submission and review process employed by some RCMI Centers
Heavy teaching load for junior faculty	Reduced teaching assignments to encourage grant writing
Insufficient protected release time for clinicians	Institutional investment in clinicians who are interested in conducting research
Limited pool of qualified applicants	Revise eligibility criteria to expand pool
Low representation of clinical researchers vs basic/behavioral researchers	Require collaboration between clinical and basic/behavioral researchers in pilot project proposals
Limited support for biostatistics and study design	Gain access to biostatistics and study design expertise across RCMI Centers
Delays in review and approval of human subjects research applications	Institutional commitment to expedite review and approval processes
Delays in review and approval of vertebrate animal research applications	Institutional commitment to expedite review and approval processes

**Table 5:** Alignment of Pilot Projects Programs.

Processes and Procedures	Outputs and Deliverables	Annual Outcomes and Goals
Develop reviewer registries of RCMI and non-RCMI investigators with subject-matter expertise and NIH study section experience Develop on-line grant submission and review systems Develop an internal grants review system	Prepare manual of best practices and standard operating procedures for Pilot Projects Programs Prepare a webinar series of presentations by pilot project awardees	Each pilot project awardee submits at least one NIH grant application Each pilot project awardee uses at least one RCMI or non-RCMI core facility Each pilot project awardee publishes at least one peer-reviewed article or presents at least one poster

**Table 6:** Expected Outcomes of Key Activities for Mentoring and Professional Development.

Key Activity	Action Plan	Key Metrics	Expected Outcomes
Provide help in preparation of NIH grant applications	Develop registry of mentors by category and NIH ICs Leverage grant writing courses and other resources	# K, SC and R grants prepared by category and NIH ICs # other grants prepared by category and NIH ICs	Overall increased submission of K, SC, R and other grant applications
Perform NIH-type review before submission	Develop Internal Grants Review System Develop registry of reviewers by category and NIH ICs	# grants reviewed by category and NIH ICs # K, SC, R and other grants reviewed	Overall increased success of K, SC, R and other grant applications
Provide training in and assist with the preparation and review of manuscripts	Develop registry of mentors by category and NIH ICs Leverage editing expertise Leverage scientific expertise for reviews	# manuscripts prepared # manuscripts accepted # manuscripts by category # manuscripts by NIH ICs that acknowledge U54	Overall increased success of high-quality papers submitted and published
Provide training in and assist with the preparation of oral and poster presentations	Develop registry of mentors by category and NIH ICs Leverage editing expertise Leverage scientific expertise for reviews	# oral and poster presentations # presentations by category and NIH ICs	Overall increased success of high-quality oral and poster presentations
Enrich mentoring milieu of early- stage RCMI investigators	Coordinate and leverage mentoring infrastructure	# postdoctoral fellows advancing to faculty # faculty promoted and/or tenured	Overall acceleration toward career advancement

Abbreviation: #, number of.

**Table 7:** Regional distribution of RCMI Centers.

Region	State	RCMI Center
Western	AZ	Northern Arizona University
	CA	Charles R. Drew University of Medicine and Science
		San Diego State University
		University of California Riverside
	HI	University of Hawaii at Manoa
Southern	AL	Tuskegee University
	LA	Xavier University of Louisiana
	MS	Jackson State University
	TX	Texas Southern University
		University of Houston
		University of Texas at El Paso
Southeastern	FL	Florida Agricultural and Mechanical University
		Florida International University
	GA	Clark Atlanta University
		Morehouse School of Medicine
	PR	Ponce Health Sciences University
		University of Puerto Rico Medical Sciences Campus
Eastern	DC	Howard University
	MD	Morgan State University
	NC	North Carolina Central University
	TN	Meharry Medical College
